# Validation of a Measure of Flipped English Learning Readiness and Examination of Its Relationships With Instructional Practices, Learning Self-Efficacy, and Learning Beliefs

**DOI:** 10.3389/fpsyg.2022.846781

**Published:** 2022-06-10

**Authors:** Shuqiong Luo, Zhengdong Gan

**Affiliations:** Faculty of Education, University of Macau, Taipa, China

**Keywords:** flipped English learning readiness, instructional practices, English learning self-efficacy, language learning beliefs, structural equation modeling

## Abstract

This study validated the Flipped English Learning Readiness Questionnaire, and examined its relationships with teacher instructional practices, English learning self-efficacy, and language learning beliefs. A total of 442 Chinese university EFL students voluntarily participated in this research. Exploratory and confirmatory factor analysis resulted in determination of five flipped English learning readiness factors (i.e., *doing previews, in-class communication self-efficacy, positive experience, intentional behaviors, and self-directed learning*) with a strong psychometric basis. The composite reliability, average variance extracted, and HTMT ratio of correlations further confirmed the convergent and discriminant validity of the Flipped English Learning Readiness Questionnaire. Structural equation modeling analysis suggested that English learning self-efficacy was a significant predictor of student flipped English learning readiness among students from different year-levels. Autonomy-supportive instructional practices significantly predicted year 2 students’ self-directed learning while grammar/translation-oriented learning had a significantly positive influence on year 3 students’ positive flipped learning experience and intentional behaviors.

## Introduction

In recent years, flipped learning has become popular (Yilmaz, 2017). In essence, flipped learning is a student-centered approach which reverses the face-to-face class time that instructors use to deliver fundamental knowledge with the out-of-class time that learners use to learn through collaborative, interactive, and problem-solving activities (Bergmann and Sams, 2012; Bicen and Beheshti, 2019). In the flipped classroom, teachers have become organizers, and facilitators (Bicen and Beheshti, 2019) who play important roles in understanding students’ learning demands and stimulating their engagement in learning activities (Hung, 2017). Numerous advantages brought by flipped language learning have been highlighted in the literature, including a better peer-assisted interactive learning atmosphere (Hsieh et al., 2017), more opportunities for individual comments (Zou et al., 2020), higher learning engagement (Hung, 2017), enhanced innovative learning strategies (Baepler et al., 2014; Chang and Lin, 2019), and better academic performance (Lee and Wallace, 2018).

Despite the merits, the flipped language learning continues to face challenges related to students’ preparation or readiness, such as students’ passive learning attitudes (Chen et al., 2014), neglect of learning materials (Thai et al., 2017), unwillingness to watch online video lectures (Lee and Wallace, 2018), and the lack of computer techniques to manage flipped learning. Insufficient readiness for flipped learning in students may result in ineffective learning, weak self-efficacy, and poor engagement (Strayer, 2012; Flumerfelt and Green, 2013; Sun et al., 2017). While the negative consequences of insufficient readiness in flipped learning have been recognized in the literature, the role of students’ readiness in the flipped English classroom has not been adequately explored particularly in the Chinese EFL (i.e., English as a foreign language) context. Although studies on educational psychology and second language acquisition have underscored the importance of teachers’ role, learning self-efficacy, and learning beliefs in the language classroom (Horwitz, 1986; Pintrich et al., 1991; Bandura, 2006; Hung, 2017), relatively little is known about how instructional practices, learning self-efficacy, and learning beliefs are associated with learner readiness in the flipped English classroom.

Given this research gap, the present research aimed to validate a flipped English learning readiness questionnaire and examine the relationships between flipped English learning readiness, teacher instructional practices, student English learning self-efficacy, and language learning beliefs among years 1, 2, and 3 Chinese university EFL students. It is believed that this research contributes to the theoretical and pedagogical discussions on the learner readiness for flipped English learning in an EFL context.

## Literature Review

### Flipped Language Learning

Flipped learning is a student-centered pedagogical approach that reverses traditional learning to independent out-of-class study (Bergmann and Sams, 2012) and combines online instruction and face-to-face learning (Shih et al., 2019). Flipped learning approach, originating from social constructivism (Bishop and Verleger, 2013), has the features of a student-centered approach that concentrates on the crucial role of social interaction in knowledge production in learning activities (Vygotsky, 1978). In the flipped learning model, more face-to-face classroom time is allocated for teachers to undertake in-class activities to contextualize and extend the learning that has previously occurred outside class (Chang and Lin, 2019); instructors can also guide students in critical thinking and monitor activities in class (Mebring, 2016). In recent years, flipped learning has been posited to provide numerous educational merits to language classrooms (Zou et al., 2020). For example, flipped language classroom can enable language learners to spend less time listening to long lectures, have more opportunities to solve problems individually and learn collaboratively with the help of peers and lecturers (Mebring, 2016; Zou et al., 2020). Additionally, the flipped language classroom allows time to be devoted to providing language learners with a dynamic learning atmosphere (Hsieh et al., 2017), improving their English reading comprehension (Lin et al., 2018), and cultivating their participation and interaction (Chuang et al., 2018).

### Learner Readiness for Flipped Language Learning

Scholars have noted that learner readiness is one of the prerequisite conditions for learners’ active participation, academic performance, and effectiveness of online learning (Dangol and Shrestha, 2019; Tang et al., 2021), which determine the success of either online learning or flipped learning (Thorndike, 1932; Yilmaz, 2017). As Yilmaz (2017) argued, students should own some responsibilities, skills, and opportunities to learn effectively in the flipped classroom. Otherwise, it would be difficult for them to participate actively in the learning activities and utilize the learning resources available (Yilmaz, 2017). Meanwhile, there have been reports of students having difficulty in flipped language learning particularly in an EFL context, such as students getting used to teacher-directed learning (Chen et al., 2014), students’ insufficient digital abilities to manage flipped learning, and student unwillingness to watch online video lectures (Lee and Wallace, 2018). Consequently, it remains unclear whether university students are ready to embrace flipped English learning, what factors may influence their flipped English learning readiness, and how we can enhance flipped English learning readiness particularly in the Chinese EFL students. Investigation into these issues can inform policy makers and curriculum designers in initiating educational interventions that intend to enhance students’ self-directed English learning with technology.

Learner readiness for online learning or flipped learning refers to personal abilities to use online learning resources and multimedia technologies to enhance individual learning quality (Kau and Abas, 2004), which represent the combination of computer self-efficacy, internet self-efficacy, online communication self-efficacy, self-directed learning, learner control, and motivation toward e-learning (Yilmaz, 2017). Following the previous studies, learner readiness for flipped English classroom in this current research is defined as learners’ preparation for the flipped English learning consisting of *doing previews* (Hao, 2016), *in-class communication self-efficacy* (Hao, 2016), *positive experience of flipped classroom* (Shih et al., 2019), *intentional behaviors* (Wu et al., 2010; Shih et al., 2019), and *self-directed learning* (Hao, 2016).

Several factors have been found in the literature to contribute to learner readiness that may intertwine with the online or flipped learning classroom (Hao, 2016; Heo and Han, 2018; Jiang et al., 2021). Preferred teacher characteristics and the utilization of the Internet were found to make a difference to secondary students’ readiness in the flipped English classroom (Hao, 2016). Hao (2016) noted that students were more motivated to do previews when they perceived their English teachers as empathetic or with a good appearance, and those who reported higher-level motivation tended to have greater readiness in technology self-efficacy. Others found that motivation and academic stress were significant predictors of college students’ self-directed learning readiness (Heo and Han, 2018). Learners’ attitudes could also positively impact learner readiness for online flipped English learning when controlling the learning tasks (Jiang et al., 2021).

### Instructional Practices

Teachers’ instructional practices play a significant role in students’ learning. According to Ames (1992), autonomy-supportive instructional practices and feedback are the crucial elements of teachers’ instructional practices that shape learners’ motivation and learning behaviors (Pintrich and Zusho, 2002). Scholars have argued that opportunities for autonomy have the potential to cultivate learners’ intrinsic interest and learning responsibility (Ryan and Deci, 2000), and that sufficient formative feedback can enhance learners’ self-regulated learning (Perry et al., 2002; Van Grinsven and Tillema, 2006). Autonomy-supportive instructional practices provide learners with chances to participate and control their learning in class (Lau, 2012). This is resonant with the self-regulated learning model in which the agency and active role of learners are acknowledged (Butler and Winne, 1995). One effective means of fostering autonomy is to encourage students to engage in cooperative learning activities (Perry et al., 2002; Van Grinsven and Tillema, 2006).

At the heart of teacher support is a teacher providing formative feedback to students in relation to a learning task (Carless, 2015). As an essential component of self-regulated learning in social cognitive theory (Schunk and Zimmerman, 1997), feedback can help students recognize the weakness and strengths of their work and control their learning (Hyland, 2000). Shute (2008) defines formative learning feedback as information communicated to learners, which is intended to modify learner thinking or behavior. Evidence from previous studies reveals that teacher formative learning feedback is instrumental in facilitating students’ language learning and involving them in self- or peer assessment (Mory, 2004; Law, 2008). In the Chinese EFL learning context, recent empirical studies confirm the positive impacts of teacher formative feedback practices on students’ learning engagement (e.g., Zhang et al., 2021).

### Learning Self-Efficacy

Self-efficacy, which is usually defined as learners’ ability to accomplish a task and the confidence in their skills to perform that task (Pintrich et al., 1991), is indispensable to learners’ aptitude, achievements, and performance (Bandura, 2006), and is strongly associated with students’ previous learning experiences (Bai et al., 2019). Self-efficacy has been shown to influence students’ learning motivation, the goals they choose to pursue, and the utilization of self-regulated strategies to perform an academic task (Linnenbrink and Pintrich, 2003; Carmichael and Taylor, 2005).

Studies in the literature have highlighted the importance of self-efficacy in student learning and performance. For example, Liem et al. (2008) reported that students with higher-level of self-efficacy were more self-regulated and more likely to regard difficulties as learning opportunities, whereas those with low self-efficacy were more likely to perceive failure as insufficient ability (Tsai, 2019). Self-efficacy has also been shown to relate to learners’ classroom participation and use of technology (Chou et al., 2010; Holden and Rada, 2011; Yilmaz, 2016). Students who had higher level of self-efficacy showed better participation in the learning activities while those with poor self-efficacy were wary of the technological advancements in the online learning environment (Yilmaz, 2016).

### Language Learning Beliefs

Learners’ language learning beliefs are defined as learners’ attitudes and ideas regarding the tasks or challenges of acquiring a second or foreign language (Kalaja and Barcelos, 2006). Research suggests that learning beliefs shape the types of technology resources undergraduate students selected to utilize and their styles of interacting with these technological resources (Lai, 2019). The more learners believed in exploring chances to apply their language knowledge in everyday life, the more they were inclined to utilize technology to supplement their language learning outside the classroom (Lai and Gu, 2011).

In the field of second language acquisition, learning beliefs have been recognized as an essential source of individual differences closely associated with language learning outcomes (Ellis, 2008). It is important to understand the role language learning beliefs play in flipped language learning. According to Boden (2005) and Hao (2016), language learning beliefs and learner readiness were closely intertwined. Boden observed (2005) that students’ learning beliefs were linked with their readiness for self-directed learning. Hao (2016) study also showed that middle school students in Taiwan with higher learning beliefs in communication strategies were more capable of self-regulating their learning in the flipped English learning context.

Previous literature suggests that whether learners are prepared for flipped learning tends to determine their learning effectiveness in the flipped classroom (Yilmaz, 2017). However, previous studies on learner readiness in the flipped English classroom were quite limited in that mainland Chinese EFL students’ flipped English learning experience has not been well-researched. Building on the research work reviewed above, this study aims to validate a measure of flipped English learning readiness and examine its relations with teacher instructional practices, student English learning self-efficacy, and language learning beliefs. Specifically, our inquiry was guided by the following two research questions:

1. To what extent is there evidence to support reliability and validity of the Flipped English Learning Readiness Questionnaire?
2. Do teacher instructional practices, student English learning self-efficacy, and language learning beliefs have significant relations with student flipped English learning readiness?

## Materials and Methods

### Research Context

The present study was situated in College English, a mandatory EFL course for Chinese non-English majors. In the flipped English classroom for non-English majors, students read learning materials, watch video lectures, and finish assignments through online learning platforms before coming to the class. When students participate in the face-to-face section of flipped English classrooms, they engage in the activities and discussions organized by the instructors.

### Participants

The sample for this study consisted of 442 undergraduates aged 18–24 years old from a first-tier university in southern China, including year 1 (*n* = 191, 43.21%), year 2 (*n* = 160, 36.19%), and year 3 students (*n* = 91, 20.6%). Males (54.3%) and females (45.7%) were roughly equally represented in the sample. The participants spanned six subject domains (design, liberal arts, science, medicine, law, and business). All the participants had learning experiences in the flipped English classroom and speak Chinese as their first language.

### Instruments

#### Flipped English Learning Readiness Questionnaire

The Flipped English Learning Readiness Questionnaire consisted of five subscales (Appendix): (1) doing previews (3 items, α = 0.74, e.g., “I enjoy doing previews by using online learning platforms”), (2) in-class communication self-efficacy (3 items, α = 0.82, e.g., “I feel confident participating in class discussion”), (3) positive experience of flipped classroom (5 items, α = 0.87, e.g., “I feel more flexible of learning time in the flipped classroom”), (4) intentional behaviors (3 items, α = 0.92, e.g., “I would like to continually use the flipped learning in my learning”), and (5) self-directed learning (3 items, α = 0.79, e.g., “I set up my own English learning goals”). Items of the Flipped English Learning Readiness Questionnaire were adapted from existing questionnaires such as: Online Learning Readiness Scale (Hung et al., 2010), Flipped Learning Readiness Scale (Hao, 2016), and Perception of Flipped Classroom Questionnaire (Wu et al., 2010; Shih et al., 2019). These existing scales were initially designed to assess learner readiness in online or flipped learning environments without focusing on English learning in university context.

#### Instructional Practices Questionnaire

The Instructional Practices Questionnaire aimed to measure students’ perceptions of their English teachers’ instructional practices in the flipped English classroom. The questionnaire contains two subscales: (1) autonomy (4 items, α = 0.83, e.g., “My teacher encourages us to interpret learning materials by ourselves”); and (2) formative learning feedback (4 items, α = 0.87, e.g., “My teacher gives clear and concrete assessment criteria for assignments or tests”). The items in the instructional practices questionnaire were adapted from Law (2008) and Lau (2012) scales that were designed to evaluate secondary school students’ perceived teacher instructional practices in the Chinese classroom.

#### English Learning Self-Efficacy Questionnaire

The English Learning Self-Efficacy Questionnaire consisted of four items (α = 0.81, e.g., “I expect to do well in this class”) used to assess participants’ learning self-efficacy in English courses. This questionnaire was adapted from Pintrich et al. (1991). A Manual for the Use of Motivated Strategies for Learning Questionnaire, which was developed to measure learners’ learning self-efficacy without focusing on a specific domain.

#### Language Learning Beliefs Questionnaire

The items of the English Learning Beliefs Questionnaire contained two subscales: (1) communication-oriented learning (4 items, α = 0.77, e.g., “I believe that the best way to learn English is to enjoy learning it”); and (2) grammar/translation-oriented learning (4 items, α = 0.69, e.g., “Mastering English means acquiring English grammar”). Items of communication-oriented learning referred to learners being interested in and gaining enjoyment from learning English, while grammar/translation-oriented learning items referred to learners being interested in grammar drill and interpretation of texts through translation in English learning (Sakui and Gaies, 1999; Yashima et al., 2017). The questionnaire was adapted from Yashima et al. (2017) which was originally used to assess Japanese learners’ English learning beliefs in an EFL context.

All the items in the above questionnaires were rated on a 5-point Likert scale, ranging from “completely disagree” (1) to “completely agree” (5). These four questionnaires were initially written in English and were translated into Mandarin since the researchers believed that presenting the questionnaires in Chinese to the participants would be more appropriate because the first language of the participants in this study was Mandarin (Gan et al., 2019).

### Data Analysis

Exploratory Factor Analysis (EFA) with the principal component extraction method and Promax rotation was performed to estimate the factor structure of the Flipped English Learning Readiness Questionnaire. Confirmatory factor analysis (CFA) was conducted to examine the construct validity of the questionnaire. The following fit indices were applied to evaluate the model fits of the CFA: chi-square statistic (χ^2^) and its degrees of freedom (df), *p*-value; the Comparative Fit Index (CFI; > 0.90 indicates good fit); Tucker-Lewis Index (TLI; > 0.90 indicates good fit); and the Root Mean Square Error of Approximation (RMSEA; < 0.08 indicates good fit) (Hu and Bentler, 1999). The convergent validity of the questionnaire was then examined through composite reliability (CR) and average variance extracted (AVE) (Hair et al., 2010). Furthermore, the discriminant validity of the questionnaire was evaluated through heterotrait-monotrait (HTMT; Henseler et al., 2015) ratio of correlations, which is utilized to assess the degree to which a particular variable’s items differ from their indicators (Sekaran and Rani, 2010).

Furthermore, Pearson product-moment correlation analysis was used to assess the correlations of flipped learning readiness variables, teacher instructional practices, student English learning self-efficacy, and language learning beliefs. Finally, to examine the structural relationships between flipped English learning readiness, teacher instructional practices, student English learning self-efficacy, and language learning beliefs, structural equation modeling (SEM) was conducted using AMOS 24.0.

## Results

### Reliability and Factor Structure of the Flipped English Learning Readiness Questionnaire

Before conducting EFA, Bartlett’s test of sphericity (Bartlett, 1954) was conducted to investigate the factorability of the data, and the Kaiser-Meyer-Olkin (KMO) test (Kaiser, 1958) was performed to measure the sampling adequacy. Results showed that the KMO coefficient was 0.896, and the χ^2^ figure of the Bartlett test of sphericity was 4466. 838 (*p* < 0.001). The KMO value above the minimum adequacy value of 0.60 and the significant Barlett test statistics indicated that the data were appropriate for factor analysis (Tabachnick and Fidell, 2007). The 17-item Flipped English Learning Readiness Questionnaire was examined through EFA using the principal component extraction method and Promax rotation, resulting in five factors with eigenvalues over 1.0 that accounted for 74.01% of the total variance.

The first factor labeled as *doing previews* included three items, accounting for 31.2% of the variance in this total scale. Factor one reflected students’ attitudes toward doing previews. The second factor, called *in-class communication* self-efficacy, contained three items, explaining 13.38% of the total variance in the questionnaire. The items of Factor Two concerned students’ self-efficacy in class discussions. The third factor was named as positive experience of flipped classroom, composing five items, accounting for 12.86% of the total variance in the questionnaire. This factor is mainly about learners’ experience in the flipped classroom. The fourth factor, term as intentional behaviors, consisted of 3 items, occupying 10.37% of the total variance in the questionnaire. The items within this factor were about students’ satisfaction with flipped learning. The fifth factor was Self-directed learning, containing three items, accounting for 6.18% of the total variance in the questionnaire. The items within this factor were related to learners’ control of their learning.

As shown in Table 1, internal consistency estimates of reliability for the five subscales in the Flipped English Learning Readiness Questionnaire are: 0.74 (doing previews), 0.82 (in-class communication self-efficacy), 0.87 (positive experience of flipped classroom), 0.92 (intentional behaviors), and 0.79 (self-directed learning), indicating good internal consistency. Item/total correlation coefficients for each subscale exceeded 0.40. The results demonstrated that the reliability of the subscales of the Flipped English Learning Readiness Questionnaire is satisfactory.

**TABLE 1:** Correlations between Flipped English learning readiness variables and instructional practices, learning self-efficacy, and learning beliefs.

	DP	CS	PE	IB	SDL	A	FLF	SE	CO	GO
DP	1									
CS	0.36**	1								
PE	0.45**	0.43**	1							
IB	0.47**	0.35**	0.83**	1						
SDL	0.32**	0.53**	0.43**	0.39**	1					
A	0.24**	0.32**	0.36**	0.29**	0.46**	1				
FLF	0.24**	0.28**	0.31**	0.24**	0.37**	0.77**	1			
SE	0.28**	0.50**	0.40**	0.31**	0.56**	0.47**	0.49**	1		
CO	0.24**	0.30**	0.36**	0.30**	0.39**	0.53**	0.49**	0.51**	1	
GO	0.21**	0.12*	0.19**	0.20**	0.12*	0.26**	0.26**	0.20**	0.37**	1

*p < 0.05; **p < 0.01.

*DP, doing previews; CS, in-class communication self-efficacy; PE, positive experience of flipped classroom; IB, intentional behaviors; SDL, self-directed learning; A, autonomy; FLF, formative learning feedback; SE, English learning self-efficacy; CO, communication-oriented learning; GO, grammar/translation-oriented learning.*

**FIGURE 1:**
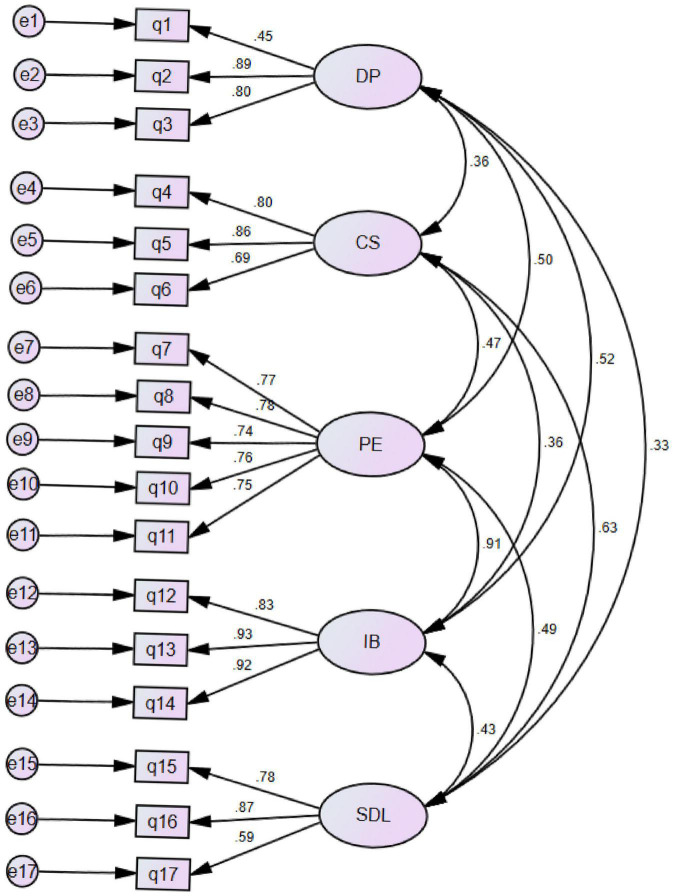
Results of the CFA, a Five-Factor Model of the flipped English learning readiness questionnaire. DP, doing previews; CS, in-class communication self-efficacy; PE, positive experience of flipped classroom; IB, intentional behaviors; SDL, self-directed learning.

CFA using the maximum likelihood estimation through AMOS 26 was conducted to confirm the factor structure of the Flipped English Learning Readiness Questionnaire identified through EFA. The assumptions of normality and absence of outliers were assessed. The fit indices of CFA suggested that the measurement model of the five subscales provided a good fit to the data, χ^2^/*df* = 2.91, *p* < 0.001; CFI = 0.95; TLI = 0.94; RMSEA = 0.066. Significant correlations existed among the five factors (*r* = 0.32–0.83). Figure 1 demonstrates the standardized results for the five-factor correlated model. In this model, all 17-item parameter estimates were statistically significant (*p* < 0.001). The standardized parameter estimates for each item ranged from 0.45 to 0.93, with all the standardized parameter estimates greater than the benchmark value 0.50 except item 1 whose value was slightly below 0.50 (see Figure 1). Cronbach’s alphas of the subscales ranged from 0.898 to 0.909, and Cronbach’s alpha of the whole questionnaire was 0.927.

To further examine the construct validity of the Flipped English Learning Readiness Questionnaire, we tested the convergent and discriminant validity. The convergent validity was assessed through the CR (acceptable if >0.60) and AVE (acceptable if > 0.50) (Hair et al., 2010). As demonstrated in Table 2, the CR values for the five factors were 0.78, 0.83, 0.87, 0.92, and 0.81 respectively, exceeding the cut-off point of 0.60, while the AVE for all the five factors exceeded the threshold value 0.50.

**TABLE 2:** Convergent validity of confirmatory factor analysis of the flipped English learning readiness questionnaire.

Measures	Items	Composite reliability	Average variance extracted
DP	3	0.78	0.57
CS	3	0.83	0.62
PE	5	0.87	0.58
IB	3	0.92	0.80
SDL	3	0.81	0.60

*DP, doing previews; CS, in-class communication self-efficacy; PE, positive experience of flipped classroom; IB, intentional behaviors; SDL, self-directed learning.*

In terms of discriminant validity, the HTMT ratio of correlations with values lower than 0.85 indicating good validity (Henseler et al., 2015) was adopted in this research. To calculate the HTMT ratio, we used the “HTMT plugin” developed by Gaskin et al. (2019). As shown in Table 3, except for the correlation between “positive experiences” and “intentional behaviors” that was slightly >0.85, all other values range from 0.398 to 0.664 (<0.85; Henseler et al., 2015). These results indicate the acceptable discriminant validity of the Flipped English Learning Readiness Questionnaire.

**TABLE 3:** HTMT Ratio of correlations among the Flipped English learning readiness questionnaire factors.

	DP	CS	PE	IB	SDL
DP	–				
CS	0.466	–			
PE	0.561	0.503	–		
IB	0.577	0.398	0.922	–	
SDL	0.42	0.664	0.518	0.456	–

*DP, doing previews; CS, in-class communication self-efficacy; PE, positive experience of flipped classroom; IB, intentional behaviors; SDL, self-directed learning.*

### Relationships Between Flipped English Learning Readiness, Instructional Practices, English Learning Self-Efficacy, and Language Learning Beliefs

Table 1 demonstrates the Pearson product-moment correlations among all the measured variables in the four questionnaires. Flipped English learning readiness factors (i.e., *doing previews*, *in-class communication self-efficacy*, *positive experience*, *intentional behaviors*, and *self-directed learning*) were positively correlated with teacher instructional practices, English learning self-efficacy, and language learning beliefs variables (i.e., *autonomy*, *formative learning feedback*, *English learning self-efficacy*, *communication-oriented learning*, and *grammar/translation-oriented learning*) (0.12 ≤ *r* ≤ 0.83 *p* < 0.01).

Structural equation modeling (SEM) was conducted to further examine the relationships between flipped English learning readiness, instructional practices, English learning self-efficacy, and English learning beliefs variables among year 1, year 2, and year 3 students respectively. The results achieved an acceptable fit for year 1 students, χ^2^/*df* = 1.81; CFI = 0.92; TLI = 0.92; RMSEA = 0.06; an adequate fit for year 2 students, χ^2^/*df* = 1.62; CFI = 0.91; TLI = 0.90; RMSEA = 0.06; and an acceptable fit for year 3 students: χ^2^/*df* = 2.07; CFI = 0.93; TLI = 0.92; RMSEA = 0.07.

**FIGURE 2:**
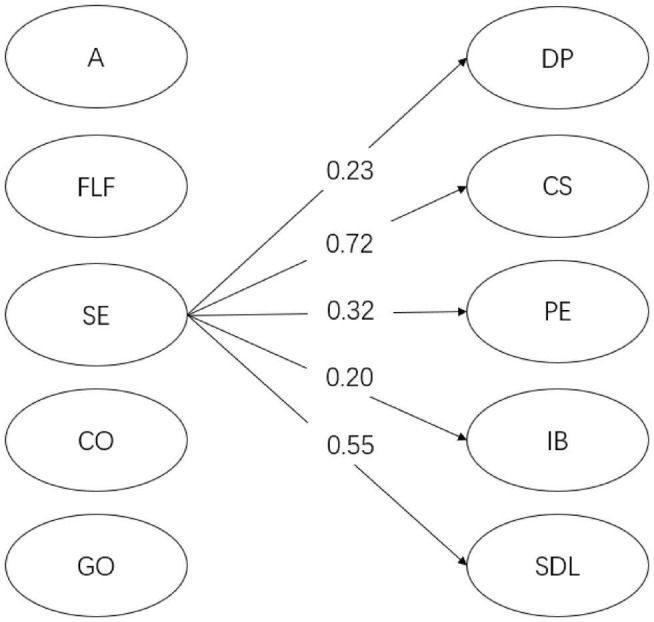
The SEM model for year 1 students. Only significant relations are presented in the figure. DP, doing previews; CS, in-class communication self-efficacy; PE, positive experience of flipped classroom; IB, intentional behaviors; SDL, self-directed learning; A, autonomy; FLF, formative learning feedback; SE, English learning self-efficacy; CO, communication-oriented learning; GO, grammar/translation-oriented learning.

**FIGURE 3:**
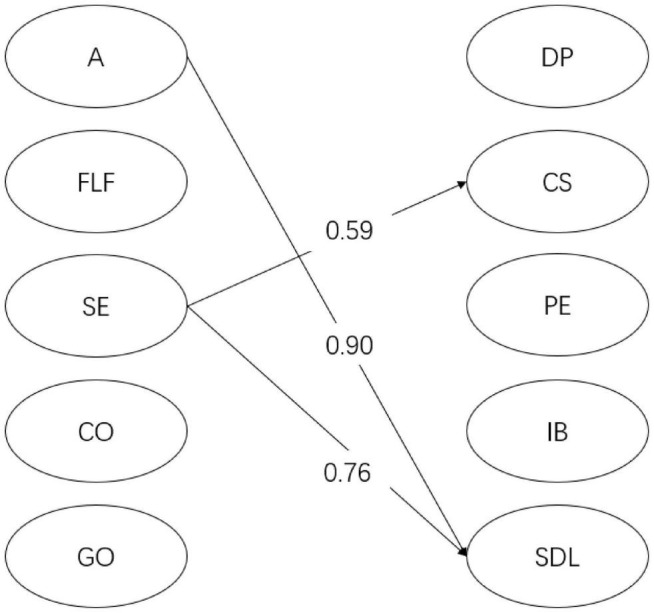
The SEM model for year 2 students. Only significant relations are presented in the figure. DP, doing previews; CS, in-class communication self-efficacy; PE, positive experience of flipped classroom; IB, intentional behaviors; SDL, self-directed learning; A, autonomy; FLF, formative learning feedback; SE, English learning self-efficacy; CO, communication-oriented learning; GO, grammar/translation-oriented learning.

**FIGURE 4:**
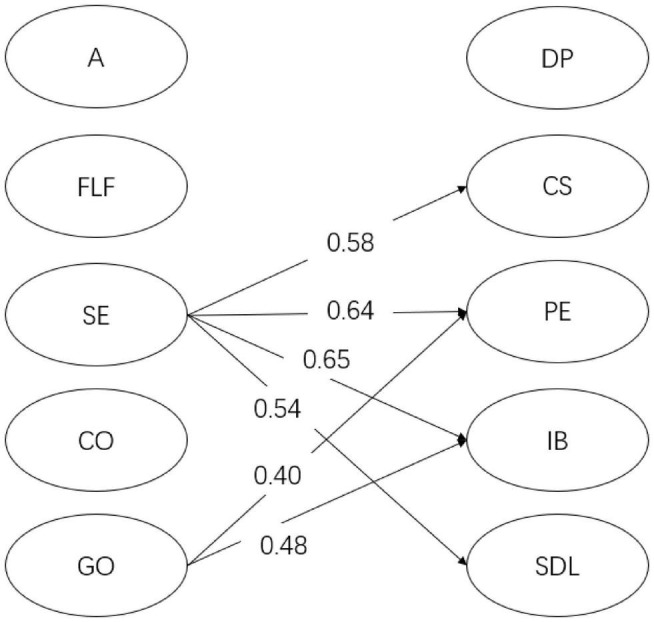
The SEM model for year 3 students. Only significant relations are presented in the figure. DP, doing previews; CS, in-class communication self-efficacy; PE, positive experience of flipped classroom; IB, intentional behaviors; SDL, self-directed learning; A, autonomy; FLF, formative learning feedback; SE, English learning self-efficacy; CO, communication-oriented learning; GO, grammar/translation-oriented learning.

For year 1 students (see Figure 2), English learning self-efficacy had a significant positive relation with *doing previews* (β = 0.23, *p* < 0.05), *in-class communication self-efficacy* (β = 0.72, *p* < 0.001), *positive experience* (β = 0.32, *p* < 0.001), *intentional behavior* (β = 0.20, *p* < 0.05), and *self-directed learning* (β = 0.55, *p* < 0.001). However, instructional practices and English learning beliefs did not show any significant relationship with flipped English learning readiness factors. As shown in Figure 3, for year 2 students, English learning self-efficacy significantly predicted the two dimensions of flipped English learning readiness, namely, *in-class communication self-efficacy* (β = 0.59, *p* < 0.01) and *self-directed learning* (β = 0.76, *p* < 0.01). In addition, autonomy-supportive instructional practices significantly predicted *self-directed learning* (β = 0.90, *p* < 0.05). No significant relationship was found between English learning beliefs and flipped English learning readiness factors. As demonstrated in Figure 4, for year 3 students, English learning self-efficacy also had a positive effect on *in-class communication self-efficacy* (β = 0.58, *p* < 0.001), *positive experience* (β = 0.64, *p* < 0.001), *intentional behaviors* (β = 0.65, *p* < 0.001), and *self-directed learning* (β = 0.54, *p* < 0.01). Grammar/translation-oriented learning positively predicted the two aspects of flipped English learning readiness, i.e., *positive experience* (β = 0.40, *p* < 0.05) and *intentional behaviors* (β = 0.48, *p* < 0.05). However, instructional practices were not significantly related to any flipped English learning readiness factor.

## Discussion

The present study validated the 17-item Flipped English Learning Readiness Questionnaire in the Chinese EFL context. The findings demonstrated that the Flipped English Learning Readiness Questionnaire has adequate psychometric properties. The EFA results have identified five dimensions of flipped English learning readiness: *doing previews, in-class communication self-efficacy*, *positive experience*, *intentional behaviors*, and *self-directed learning*. The first factor, *doing previews*, is linked to the FELR principle, which means students utilizing technology to read or watch learning materials before class (Hao, 2016). In this dimension, the sampled university students seemed to enjoy finishing preview assignments by reading learning materials, watching online lectures, and using online learning platforms. The dimension of *in-class communication self-efficacy* links to the essence of FELR as a knowledge acquisition procedure engaging learners’ ability and confidence to communicate with instructors or peers in class. The sampled students appeared to be confident in expressing their ideas and raising questions in class discussion. The third factor, *positive experience* of flipped classroom, is related to the FELR approach that encourages an interactive learning atmosphere for individual support or feedback from instructors (Zou et al., 2020). This study showed that the sampled students were satisfied with the flipped English classroom, pointing to the advantages of the flipped English classroom for the interactive and collaborative learning atmosphere and opportunities for feedback and support (Hsieh et al., 2017; Zou et al., 2020). The dimension of *intentional behaviors* is associated with learners’ satisfaction in the flipped classroom (Shih et al., 2019). We postulate that the students who scored high on this factor would be more likely to continue flipped English learning. The fifth factor, *self-directed learning*, highlights the aspects of FELR that students can direct their own learning experience and process. Those students who scored high on this factor were likely to be good at setting up their learning goals, working hard to achieve them, and seeking assistance when facing problems.

The results from CFA have confirmed the existence of the five-factor model of flipped English learning readiness in Chinese EFL students. All the constructs displayed adequate reliability, convergent and discriminant validity, suggesting that the Flipped English Learning Readiness Questionnaire is a reliable and valid measure that can be applied in assessing Chinese university EFL students’ readiness for flipped English learning in the classroom.

This study indicated that the participants’ general flipped English learning readiness was satisfactory. The relatively high mean score on *positive experience* in this study corroborated Hao (2016) finding that EFL students generally demonstrated a high level of positive experience in the flipped English classroom. This result can be attributed to the features of flipped learning, such as providing an interactive and collaborative learning environment (Zou et al., 2020) and helping learners apply their newly acquired knowledge from pre-class activities into their in-class activities effectively (Shih et al., 2019). In the current study, we also noted that students reported similar mean scores on *intentional behaviors* and *self-directed learning*. One possible explanation could be that self-directed learning skills induce the refinement of learners’ satisfaction (Yilmaz, 2017), which apparently suggests that the higher mean score on *self-directed learning*, the higher mean score on *intentional behaviors*.

Note that students’ positive response to *doing previews* and *in-class communication self-efficacy* in this study contradicts Hao (2016) finding on these two factors in his study with middle school students. It is likely that compared with secondary students, university students may have greater inclination toward doing assignments before class, expressing their ideas, and may be more willing to discuss with peer classmates.

SEM results showed that English learning self-efficacy significantly predicted all flipped learning readiness factors in year 1 students, two flipped learning readiness factors (i.e., *in-class communication self-efficacy* and *self-directed learning*) in year 2 students,’ and four flipped learning readiness factors (*in-class communication self-efficacy, positive experience, intentional behaviors, and self-directed learning*) in year 3 students. These findings provided empirical support for the predictive role of English learning self-efficacy in flipped English learning in Chinese university EFL students. The finding coincides with past studies in the literature suggesting that English learning self-efficacy was a powerful predictor of second or foreign language learning strategies (Waller and Papi, 2017; Bai, 2018; Bai and Wang, 2020).

With regard to the two instructional practices variables, only autonomy-supportive instructional practices was found to significantly predict one flipped learning readiness factor (i.e., *self-directed learning*) in year 2 students. This result is consistent with past studies that evidenced the effect of teachers’ instructional practices on shaping learners’ learning behaviors (Pintrich and Zusho, 2002) and affecting flipped English learning readiness (Hao, 2016). Similarly, with regard to the two language learning beliefs variables, only grammar/translation-oriented learning significantly predicted two flipped learning readiness factors (i.e., *positive experience* and *intentional behaviors*) in year 3 students, which resonates with the observation that students’ language learning beliefs predicted their readiness in the flipped English classroom (Hao, 2016). It could be that university EFL classroom has been usually dominated by practice activities aimed at helping students to master grammar knowledge and translation skills (Yashima et al., 2017). Taken as a whole, these results suggest that unlike English learning self-efficacy, teacher instructional practices and learner language learning beliefs did not become a consistently significant predictor across students of different year-levels.

One possible reason for the lack of a consistently predictive effect of teacher instructional practices and learner language learning beliefs in this study is the controlling of shared variance with the other variables such as English learning self-efficacy. As can be seen in Table 1, correlation analysis showed that all teacher instructional practices and learner language learning beliefs variables were positively correlated with flipped English learning readiness variables. When these teacher instructional practices and learner language learning beliefs variables and English learning self-efficacy entered into the equation together, the correlation decreased.

## Conclusion and Implications

This study has made a theoretical contribution by conceptualizing students’ flipped English readiness as having five separate dimensions and by empirically testing this theoretical assumption in a sample of Chinese university EFL students. In practice, teachers can develop an understanding of levels of student readiness for flipped English learning through utilizing the Flipped English Learning Readiness Questionnaire, which allows them to map out guidance for efficient flipped learning and teaching activities in the English classroom.

English learning self-efficacy stood out as a significant predictor of university students’ flipped English learning readiness. This finding highlights the crucial role of English learning self-efficacy in flipped English learning. As such, it will be useful for instructors to provide learners with encouragement to reinforce their confidence and sense of accomplishment in the learning process (Liem et al., 2008). It is recommended that teachers allocate sufficient flexibility for learners to complete assignments. For example, individual presentations of the same topic in different forms, such as role-playing and formal presentation can be allowed in light of student’s personality or English proficiency levels. In doing so, students’ English learning self-efficacy can be greatly enhanced, which in turn may benefit their readiness for flipped English learning.

Given the positive effect of autonomy-supportive instructional practices on self-directed learning documented in this study, we believe that teacher use of authentic learning materials (Lee et al., 2018), sincere encouragement, and guidance on student learning strategies will facilitate learners’ self-directed learning in flipped English learning. This means that, for example, the teaching of grammar and vocabulary should involve use of authentic materials, and should be integrated into communication activities (Yashima et al., 2017). We also suggest that institutions organize professional workshops, seminars, or training programs relevant to the design of flipped English learning for university English teachers, which in turn will help to enhance students’ readiness for flipped English classroom.

Several limitations of this study should be noted. First, as a cross-sectional study, the causal relationships between students’ flipped English learning readiness, instructional practices, learning self-efficacy, and learning beliefs were examined using only quantitative methods. Future studies may consider adopting experimental or longitudinal design to systematically examine the causal relationships between flipped English learning readiness, instructional practices, learning self-efficacy, and learning beliefs. Second, data were collected through self-reported survey tools in this study, which might raise issues associated with data quality and validity (Fan et al., 2006). For example, responses obtained from self-reported questionnaires might not accurately reflect participants’ true thoughts (Teo et al., 2017; Shih et al., 2019). Future research is expected to employ qualitative methods, such as individual interviews, students’ diaries, and teachers’ field-notes to investigate learner readiness more deeply. Third, the enrolled participants in this research only came from one public university in China. Consequently, the findings of this study may not be generalized to represent all university students in China. Replication in future research with larger and more representative samples is thus recommended. Finally, the current study did not look at how learner English proficiency might impact on student readiness for flipped English learning. Future research thus needs to investigate the impact of students’ English proficiency on their reaction to flipped English teaching.

## Data Availability Statement

The raw data supporting the conclusions of this article will be made available by the authors, without undue reservation.

## Ethics Statement

The studies involving human participants were reviewed and approved by the University Ethics Assessment Committee. The patients/participants provided their written informed consent to participate in this study.

## Author Contributions

SL and ZG conceived to the idea and developed the materials. SL carried out the data collection and took the lead in writing the manuscript. ZG provided the critical feedback. Both authors read and approved the final manuscript.

## Conflict of Interest

The authors declare that the research was conducted in the absence of any commercial or financial relationships that could be construed as a potential conflict of interest.

## Publisher’s Note

All claims expressed in this article are solely those of the authors and do not necessarily represent those of their affiliated organizations, or those of the publisher, the editors and the reviewers. Any product that may be evaluated in this article, or claim that may be made by its manufacturer, is not guaranteed or endorsed by the publisher.

## Appendix

### Flipped English Learning Readiness Questionnaire

(1) **Doing Previews**
  1. I enjoy doing previews by reading learning materials.
  2. I enjoy doing previews by using online learning platforms.
  3. I enjoy doing previews by watching online videos.
(2) **In-Class Communication Self-efficacy**
  4. I feel confident expressing myself in English classroom.
  5. I feel confident participating in class discussion.
  6. I feel confident in posting questions in online discussions.
(3) **Positive Experience of Flipped Classroom**
  7. I am in favor of participating in the flipped learning activities.
  8. I feel I learn better in the flipped classroom.
  9. I feel more flexible of learning time in the flipped classroom.
  10. I can get support from cooperative learning and group work with other participants in the flipped physical class.
  11. I can easily get counseling and support by the tutor in the flipped physical classroom.
(4) **Intentional Behaviors**
  12. I am satisfied that the flipped classroom meets my needs in terms of learning.
  13. I would like to continually use the flipped learning in my learning.
  14. Overall, I feel satisfied with flipped pedagogy.
(5) **Self-directed Learning**
  15. I set up my own English learning goals.
  16. I carry out my own English study plan.
  17. I seek assistance when I face problems in learning English.
